# Cypress (*Taxodium*) Knee Seasonal Growth Is Stimulated by Flood Water Levels and Constrained by the Tree Dormant Season: A 14‐Year Study

**DOI:** 10.1002/ece3.73853

**Published:** 2026-06-15

**Authors:** Maureen S. Bonness

**Affiliations:** ^1^ Boondocks Botany LLC Naples Florida USA

**Keywords:** cypress knee, pneumatophore, root dormancy, seasonal growth, starch storage, *taxodium*

## Abstract

Cypress knees are woody root structures of cypress trees (*Taxodium* spp.) that curiously protrude upwards out of the ground. To better understand knee growth characteristics, 30 knees in a southwestern Florida depressional cypress swamp were measured monthly for 14 years (2012–2025) for tip growth. Substantial knee growth, when it occurred, was during the months of June through September, but only when the month coincided with water levels above ground. Regardless of water levels or the canopy's leafiness, knee growth rates consistently declined in October, and knee growth was rarely detectable from November through March. In other words, seasonal growth of cypress knees was triggered by above‐surface water levels occurring during the tree's typical growing season. These conclusions are further supported by extreme weather years when knees failed to grow during flood‐less summers (2012, 2023), and knees remained dormant during unusual January/February wet conditions (2016, 2024). Growing seasons with higher water levels and longer hydroperiods were positively correlated with greater cypress knee tip growth, as well as a higher percentage of knees growing that season. Considering that 90% of knee tips were above mean high water at the onset of the study, the advantage of expanding a knee's thin‐barked growing tip during high water conditions may be, in addition to gaining height, to augment gas exchange with the inner tissues of the knee where aerobic respiration aids in starch storage processes and phloem's distribution of oxygenated resources. For this sample of knees, the mean seasonal growth rate was 0.39 cm/year, and a single‐season individual maximum was 3.6 cm/year.

## Graphical abstract

Data from monthly monitoring of thirty cypress knees in a southern Florida cypress dome over 14 years demonstrated that knees have a distinct dormant season from November through March, and during their growing season, knees grew only when the cypress dome had surface water. The magnitude of knee tip growth was positively correlated with longer hydroperiods and higher water levels—regardless of the fact that most knees already had tips above high water at the onset of the study. The research suggests that fresh expansion of knee tip bark during episodes of root system inundation may have benefits other than growing taller, perhaps including facilitating gas exchange.

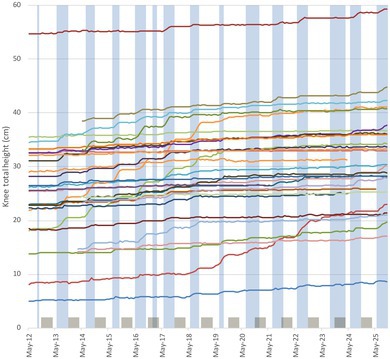

## 1 Introduction

Cypress (*Taxodium*) knees are mavericks within the world of roots. They exit a roots' typical subterranean habitat to grow above ground in an airy world that is fraught with risks that roots rarely encounter, such as fire and physical damage from falling/floating branches or animal trampling (and more recently, bulldozers, hiking boots, and boat hulls). Knees appear to develop in response to cues and hormones for negative, rather than positive, geotropism. In freshwater swamps, only *Taxodium* and its rare Asian cousin, *Glyptostrobus*, have evolved the hormone/genetic combination to create woody root structures that can be sizeable cone‐shaped monuments. Knees are unique structures that develop via secondary growth (lateral meristematic girth growth), as a bulge and expansion on the upper side of a lateral root. This contrasts with branches that develop via primary apical meristematic growth. Perhaps the botanical structures most similar to cypress knees are the “knee pneumatophores” of several *Brugueria*, *Ceriops*, and *Xylocarpus* mangrove species (Tomlinson 1986). Several tree species (e.g., *Nyssa* spp.) that grow in swamps with *Taxodium* develop “root loops” that obliquely arch above ground via primary tip growth. Apogeotropic roots have also been recorded for multiple tropical tree species (Sanford 1986; Reddell et al. 1996), but these are primary growth roots that extend upward on nearby tree trunks, do not loop back to the ground, and are generally associated with nutrient acquisition.

Cypress trees are themselves oddities, being one of the few deciduous conifers. Cypress historically dominated freshwater swamps of the southeastern United States to the extent that during the cypress timbering heyday of the early 20th century, over a billion board feet of cypress were milled in a single year (Williston et al. 1980; Mattoon 1915). Cypress trees are at their competitive best in swamps seasonally inundated for 6–10 months (Duever and Roberts 2013). There are two species of *Taxodium* ( *T. distichum* and *T. ascendens* ) with overlapping ranges in the southeastern United States. Both *Taxodium* species grow knees, but the species distinction (which can be difficult) is not pertinent for this research on cypress knee growth characteristics.

Knees represent a significant energy investment by cypress trees, with knee densities having been estimated as high as 10,000/ha (Brown 1981), thereby implying that knees provide a benefit to cypress trees. That benefit (i.e., function) has been an unresolved topic for an uncomfortably long time and has been aptly referred to as an “enduring enigma” (Briand 2000). Proposed functions include gas exchange (pneumatophores), carbohydrate storage, and mechanical support (see discussions in Briand 2000 and Rogers 2021). As secondary functions, knees may also participate in detritus collecting, methane release (Pulliam 1992), and nutrient acquisition via stump recycling (Kummer et al. 1991; Bonness 2011). The involvement of cypress knees in methane emissions has amplified attention to knee gas exchange for the purpose of calculating carbon budgets within cypress swamps (Martinez et al. 2024).

During the long history of cypress knee research, experimental studies and hypotheses have alternately supported, then refuted various knee roles. Initial presumptions were that knees served as pneumatophores based on evidence that includes: (1) knees generally grow to a height above high water, (2) cypress trees growing in upland conditions lack knees, and (3) the subsequent death of cypress trees after their knees are permanently inundated (Shaler 1883; Shaler 1887). However, the absence of aerenchyma tissue within cypress knees (Penfound 1934), along with physiological studies that demonstrated knee gas exchange was unremarkable (Kramer et al. 1952), eroded confidence in the pneumatophore hypothesis. Nonetheless, Martin and Francke (2015) completed a laboratory demonstration of oxygenation of inundated root segments when connected to above‐water knees. Also, Rogers (2021) used anatomical studies to revive Shaler's (1883) hypothesis of oxygenation of the sap and proposed a mechanism in which air filters through the bark of the knee, then oxygenated metabolites (etc.) are pumped beyond via phloem. An alternative hypothesis for knee function is mechanical support, which implies that the knee serves as a tension spring between a lateral root and vertical roots that descend below the knee (Lamborn 1890; Mattoon 1915), although there is no apparent reason why a tension spring would grow to a height roughly corresponding to high water. For the proposed function of carbohydrate storage, there have been repeated demonstrations that there is an abundance of starch within knees, but growing a woody root structure above ground for this purpose is botanically unconventional. Starch storage has been, more or less, relegated as a “fall‐back” function after other proposed functions are discarded (e.g., Brown and Montz 1986).

Knees, being woody structures, are relatively slow‐growing, and their longevity is likely underappreciated. I conducted knee growth rate studies in old‐growth cypress at Corkscrew Samp Sanctuary (Florida, USA) in permanent ecological research plots established in 1981. By revisiting those plots decades later, 70 of the tagged knees were identified and remeasured. The average growth rate for those mature knees, over the span of 32 years, was 0.2 cm/year (Bonness et al. 2013). While the maximum long‐term rate for an individual knee was 0.8 cm/year, 15% of knees were static and grew less than one cm total in 32 years. The slow knee growth rate raised the notion that some knees may be nearly as old as the cypress trees above (centuries‐old, at this site).

**FIGURE 1:**
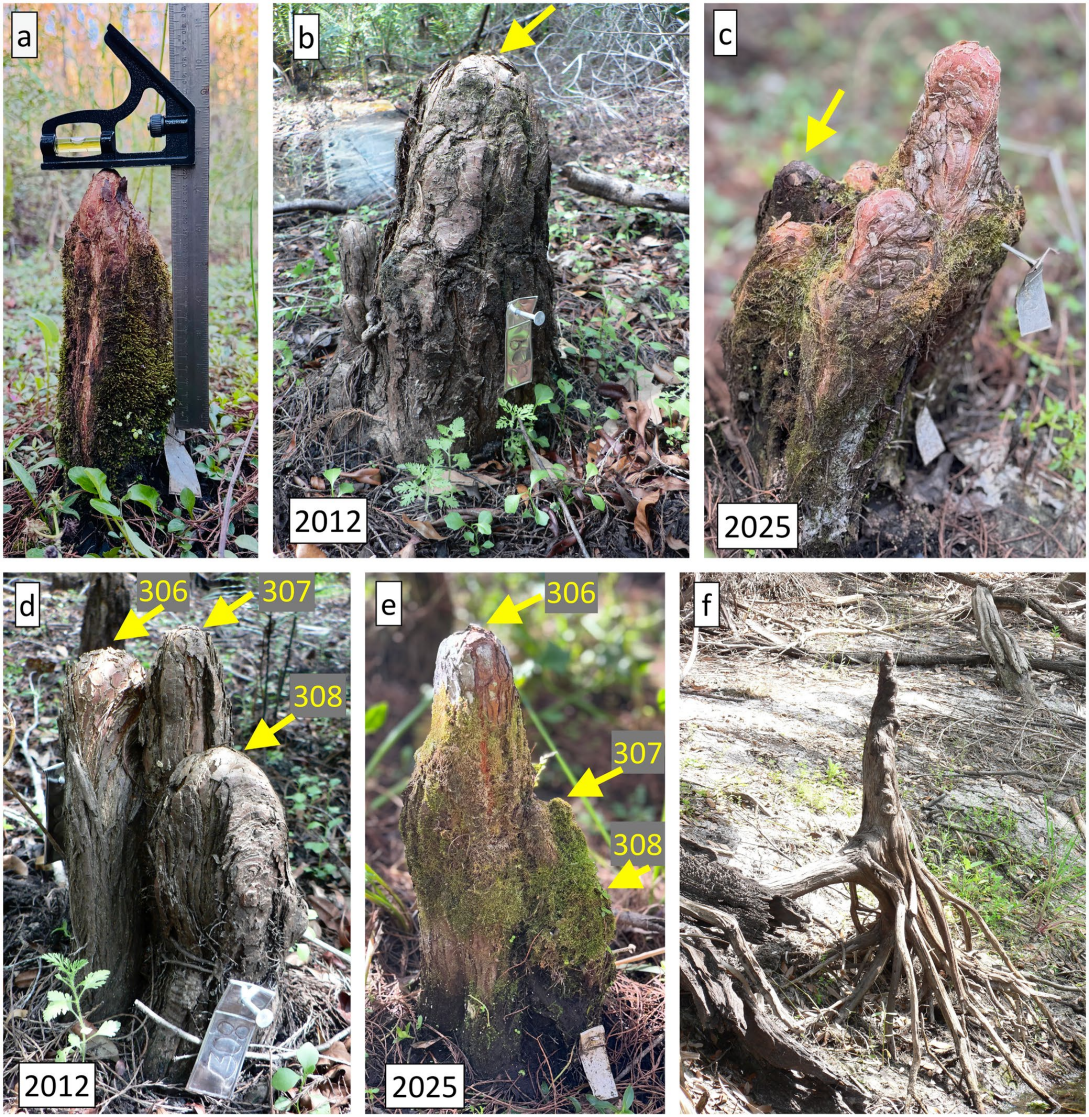
(a) Knee 304 with combination square tool for nail‐to‐tip measurement. (b) Knee 309 (arrow) in 2012, before a root loop grew over knee 309 (root loop first noted in 2019, which then surged and developed multiple tips). (c) Knee 309 (arrow) in 2025 was considered dead in 2024. The new knee (tagged #309b) above the root loop grew 9.0 cm in 2022–2025 and is not included in data analyses for this study. (d, e) Knees 306, 307, 308 (arrows L to R) in 2012 and 2025. Knee 306 surged with the fastest knee growth in the study, while knees 307 and 308 barely grew and are now mossy humps on the side of 306. (f) Anchoring roots below a knee exposed by erosion (Fisheating Creek, FL).

These data elicited questions about the growth rates of knees elsewhere. An alternative study plot was established in a depressional cypress dome near Corkscrew Swamp, with the objective of potentially documenting growth rates of faster‐growing knees. An additional objective for the cypress dome plot research was to address the question of seasonal knee growth. Longitudinal sections and cross sections of knees show ring patterns, implying seasonal growth. As knees are notoriously variable, a population of knees within the cypress dome was selected to monitor monthly changes in growth (Figure 1a). It soon became apparent that the water level was a pivotal character of the story, and that a single year of monitoring was insufficient. The hypotheses for this study were (a) the mean growth rate for knees within this cypress dome population exceeds that of knees from Corkscrew Swamp's old‐growth trees, (b) knee growth is seasonal, with parameters of the knee growing season to be determined, and (c) (hypothesis added after 18 months of data) knee growth rates are correlated with surface water levels. This study is unique among cypress knee research in following the incremental development of knees for a protracted period. In the long run, a better understanding of cypress knee ecology may contribute to insight into functionality.

## 2 Methods

### 2.1 Study Site

**FIGURE 2:**
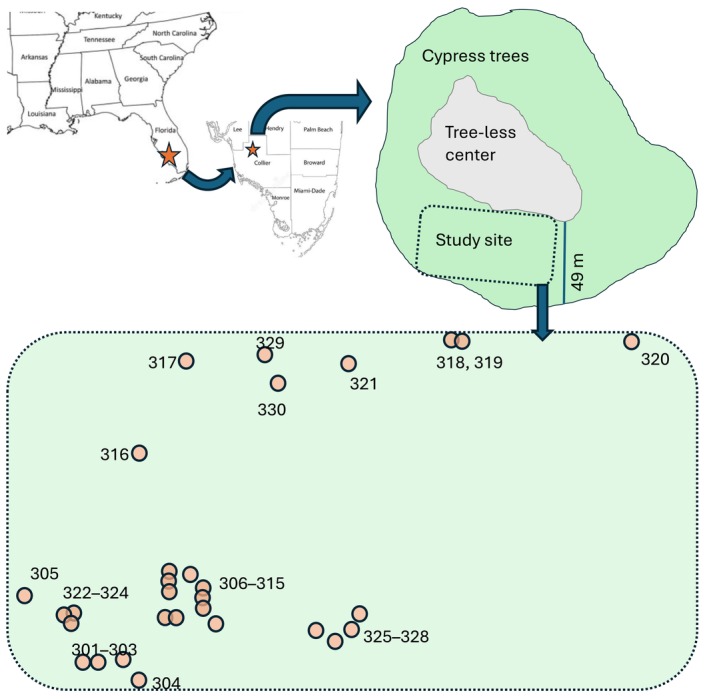
Schematic map of knees, study site, and cypress dome location.

The survey site was a cypress dome on private property adjacent to Audubon's Corkscrew Swamp Sanctuary (CSS) in Collier County, Florida, USA (Figure 2). The cypress dome is relatively undisturbed and has apparently never been cleared, although it is embedded within a neighborhood of sparse housing and some agricultural activities. During the survey period, there were no major neighborhood changes resulting in alterations to the hydrology of the study site. The dome is approximately 6 acres (2.5 ha) in size, and the surveyed knees are within an area of approximately 0.8 acres (0.3 ha). The center deep area of the dome is treeless. Larger cypress trees closer to the center have trunks greater than 40 cm DBH and are growing in peat approximately 135 cm deep. From the inner dome to the outer edge, the circumference of cypress trees decreases to approximately 25 cm DBH, and the peat depth grades to 20 cm at the outer trees. The study site's water levels are somewhat impacted by a road and ditch nearby.

### 2.2 Knee Measurements

In June 2012, 24 knees were selected for this study, and an additional six knees were added in 2014. Knee selection was predicated on the knee appearing to have recent growth characteristics, such as an orange tip or expanding bark on the tip. A nail and tag were attached to each knee. Monthly measurements were made of the distance between the top of the nail shaft and the tip of the knee. Measurements were made with a carpentry combination square, which provided a tight 90‐degree angle between a vertical ruler (placed on the nail shaft) and the sliding horizontal cross bar placed on the knee tip, with an incorporated level to ensure repeatable placement (Figure 1a). Efforts were made to provide an accuracy of 0.1 cm for monitoring changes in these slow‐growing woody structures. Precision was limited by the measuring device and affected by minor aberrations in tip bark (e.g., peeling, curling), and potentially also affected by hygroscopic swelling of the woody knee structure. Knee tip appearance was also recorded, including tip color, evidence of expanding bark, lichen, moss, etc. The total knee height (from peat to knee tip) was recorded on the first date of measurement for each knee. Several times throughout the study, measurements were made between each knee's nail shaft and water level, in order to extrapolate data for knee height above/below water during wet season conditions. From these data, it was calculated and field‐verified that knee #320 was located at the lowest elevation.

### 2.3 Water Data

Hydrologic data was sourced from the survey site as well as from the CSS database. At the study site, starting in 2014, measurements were made between the water level and a nail in a tree located in a low‐elevation area of the plot. Water measurements were recorded monthly on the same dates as knee measurements. The study site gauge was labeled such that 0.0 on the gauge was the minimum water level for surface water to occur at knee #320, the lowest elevation knee. CSS water levels were measured daily at the B Gauge, a well located in a deep pond along the Sanctuary boardwalk. The CSS border is approximately 100 m distant from the study site, and the B Gauge is 1000 m from the study site.

Calibration between the two datasets was done by comparing water levels at both sites on 60 separate dates throughout 2014–2025. Two conversion factors between the datasets were calculated, one for the high‐water season and one for the early season, while the CSS deep‐peat pond is filling. The high‐water conditions, which included all data collection dates in which the CSS B Gauge was > 80 cm (*n* = 47), provided a conversion factor of 80.3 (i.e., when the study site water gauge was at 0.0, the B Gauge registered 80.3). This was calculated as the mean difference between the two datasets on 47 dates. The high‐water conversion factor was employed when determining annual water levels. The early season conditions, which included dates when the CSS B Gauge was 65–90 cm during the first 3 months of the season (*n* = 17), had a mean difference (conversion factor) of 72.0. The early season conversion factor was employed to estimate the onset of hydroperiods for years when field data did not specify a start date (2012, 2019, and 2022). For this publication, using the conversion factors, CSS data were then re‐labeled so that 0.0 was equivalent to the study site gauge 0.0 (i.e., minimum water level for surface water at knee #320). The lowest water measurement at the study site gauge was −5 cm (i.e., water level 5 cm below ground at knee #320), and the CSS data lower limit was capped at −5 cm for consistency within analyses.

### 2.4 Data Analysis

Data were entered in Microsoft Excel to calculate summary data and population metrics. Mean annual growth for all knees over the duration of the study was calculated, as was annual growth for individual knees. Calendar month growth for all knees combined is reported. To further characterize population‐level growth patterns, the percent of knees “growing” was determined using the threshold growth rate of ≥ 0.3 cm/year, a rate that is close to the median growth rate and reflects biologically meaningful elongation. Linear regression analysis was applied both to seasonal (April 1–October 31) water levels and mean growth of all knees, and seasonal water level to percentage of knees growing. Both mean annual growth and percent of growing knees were also examined with a linear regression to the estimated hydroperiod.

Although these data could be viewed as repeated measures, the experimental design made no effort to distinguish among different sampling sites, where the issue of independent measures would affect the power of the analysis. Townend (2002) recommends repeated measures as an appropriate design for examining changes through time, but indicates the nature of the repeated measures is not relevant when summary data (i.e., mean growth of all knees, or mean growth over 14 years for a given knee) are used, as is the case in these analyses.

Nevertheless, mixed‐effect models, which account for repeated measurements of individual knees through time, were developed. These analyses were conducted with R statistical software v.4.5.3 (R Core Team 2026) using mixed‐effects models within the *lme4* package. Linear mixed‐effects models were employed to analyze the relationships between mean annual individual knee growth and hydrologic conditions (hydroperiod or seasonal mean water levels) as fixed effects, and knee identity as a random effect (intercept) to evaluate variation among knees. Models were fit by Residual Maximum Likelihood. *p*‐Values for annual growth models were calculated by the Likelihood Ratio Test. Water data from both CSS and the study site databases were included.

Mixed‐effect models were also utilized to evaluate the influence of flooding and seasonal dormancy on the occurrence of knee growth from monthly data. Generalized linear mixed‐effect models were used with a binomial error distribution, fit by Maximum Likelihood using *glmerMod* (Laplace Approximation). Flooding (the presence of water above ground surface), seasonal dormancy (November through March), and their interaction were fixed effects, while knee identity was a random effect within the models. The presence or absence of growth was the binomial response variable. For these event‐based models, a monthly growth occurrence was defined as an increase of at least 0.1 cm in the nail‐to‐tip measurement that was sustained for at least three months. Subsequent growth occurrences were recorded only if the nail‐to‐tip measurement had increased since the prior growth record, thereby minimizing noise from measurement error and seasonal shrinkage. The presence of surface water was determined by the study site water gauge for April 2014–October 2025, CSS data for June 2012–March 2014 on the same date as the knee measurement, and field notes about the onset of surface water at the study site.

## 3 Results

A total of 30 cypress knees in a depressional cypress dome were tagged and measured monthly from 2012 through 2025, although six of the knees were not included until 2014. Knees were selected based on having characteristics of recent growth (a knee tip with at least a hint of orange or splitting bark). Three of the knees died during the study (one in 2021; two in 2024). There was no apparent cause of death, with no change in bark appearance until fungal fruiting bodies emerged. In 2025, a fourth knee was retired from the study because the tip died when bark was sloughed off by a falling branch. If knee tip bark is damaged, that tip generally does not repair and regrow, although further growth of the knee may occur where bark is intact, with the sidewalls bulging out to form shoulders and new tips. In total, 398 “knee seasons” (the number of knees multiplied by the number of years) were measured over the course of 14 years.

The selected knees exhibited much architectural variability, with some knees morphing dramatically over the years (Figure 1b–e) and others relatively unchanged by time. Appendix S1 includes time‐lapsed photos of 21 knees in 2012 and 2026. Knee height, from peat to tip, ranged from 5 cm to 55 cm at the start of the study, and the average height was 28 cm. Knees closer to the dome center were up to 12 cm lower in elevation than knees further out. The mean high water level at the study site water gauge during wet season months (measured at the end of July and August, except in 2023; *n* = 22) was 15 cm. The highest water level recorded was over 35 cm after Hurricane Irma in 2017. In 2025, the average knee height above mean high water was 21 cm, with one knee tip (#301) being below water and the tallest knee tip (#320) protruding 49 cm above high water. Half of the knees were a classical cone shape, although some of the cones were merged to one or more other knees at the base, and some eventually grew secondary tips or “shoulders”. Other shapes included grouped knee conglomerates, multi‐tip knees, and knees on root loops. An estimate of knee density at the study site, based on sampling eight 50 m^2^ plots, was 5100 knees/ha with a range of 1800/ha to 12,600/ha. The underground connectedness or relatedness between tagged knees, or between knees and nearby cypress trees, is unknown.

**FIGURE 3:**
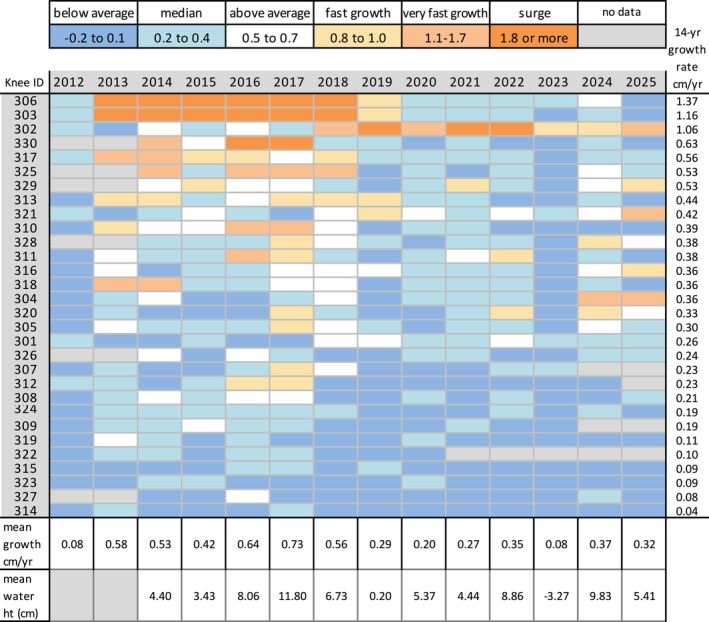
Individual knee growth variability 2012–2025. The annual growth increment (cm) for each knee is presented as color‐coded shading for relative growth rates. Individual knees (rows) are sorted by greatest to least growth rate. Annual growth was calculated by the change in knee height between January and December of each calendar year. The color bars demonstrate variability between knees and how individuals themselves vary over the years. “No Data” cells for 2021–2025 represent knees that died. “No data” cells for 2012 and 2013 are knees that were added to the study in 2014. The far right column is the 14‐year growth rate for each knee. The second row from the bottom is the mean annual growth rate for all 24–30 knees. The final row is the mean seasonal water height at the study site for April through October for each year.

There was much variability in knee growth rates between individuals, between years, and individuals themselves varied over the years. A schematic representation of individual growth rates over 14 years is presented in Figure 3. General metrics of knee growth rates are provided in Table 1. The mean growth rate for all knees over the 14 years was 0.39 cm/year. Despite surveyed knees being initially selected based on having growth potential, six knees (20%) had continuous low growth with an average of < 0.12 cm/year. Twelve knees (40%) went through growth spurts (1.0 cm/year or higher) lasting 1–8 years. None of the knees grew at above‐average growth rates (> 0.5 cm/year) for more than eight years in a row. The maximum growth rate for a knee during a single season was 3.6 cm/year.

**TABLE 1:** Summary growth rate statistics for 30 knees over a 14‐year time span (2012–2025).

14‐year growth metric	cm/year
Mean individual growth rate	0.39
Median individual growth rate	0.34
Fastest growing knee	1.37
Slowest growing knee	0.04

Due to initial knee selection criteria, the surveyed knees within this study may have a somewhat greater propensity for tip growth than the average knee in this depressional cypress dome. To better characterize this discrepancy, in August 2025 a spot check for visibly growing knees was done of all knees within 3 m of tagged knees. Sixty percent of tagged knees (*n* = 26) exhibited growth characteristics, as compared with 44% of adjacent knees (*n* = 129) showing signs of active growth.

**FIGURE 4:**
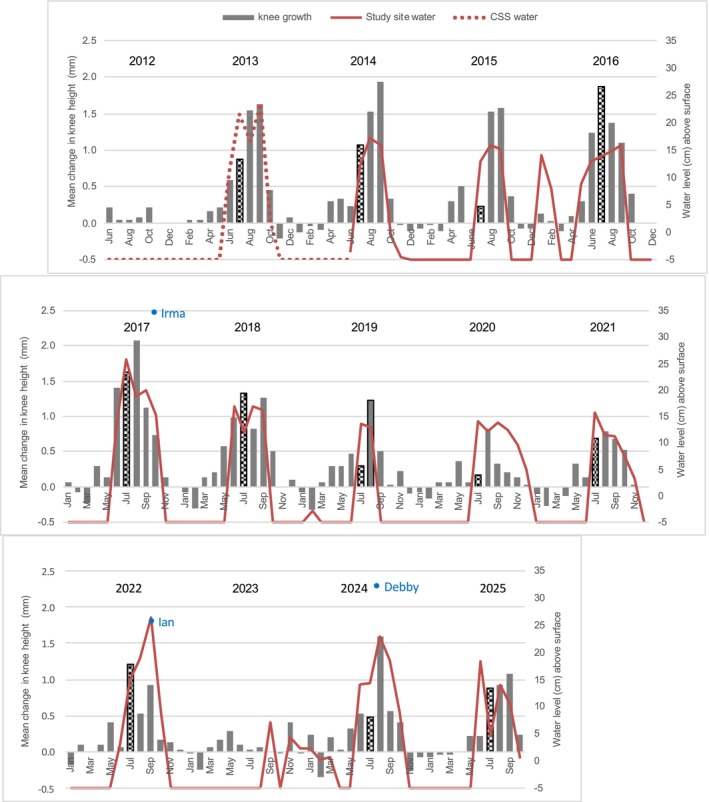
Monthly change in cypress knee height and water level 2012–2025. Each bar for knee height is the average of 24–30 knees. For each year, the July knee data bar is patterned to visualize early or late season variability. Water levels from the study site were labeled such that zero equates to the minimum for surface water at experimental knees. Study site and CSS water level measurements were taken on the same date as the monthly knee measurement. For 2012 and 2013, water levels are from CSS B Gauge with a conversion factor of 80.3. Blue dots with names represent water levels 4 days after Hurricane Irma (2017), 5 days after Hurricane Ian (2022), and 1 day after Hurricane Debby (2024).

**FIGURE 5:**
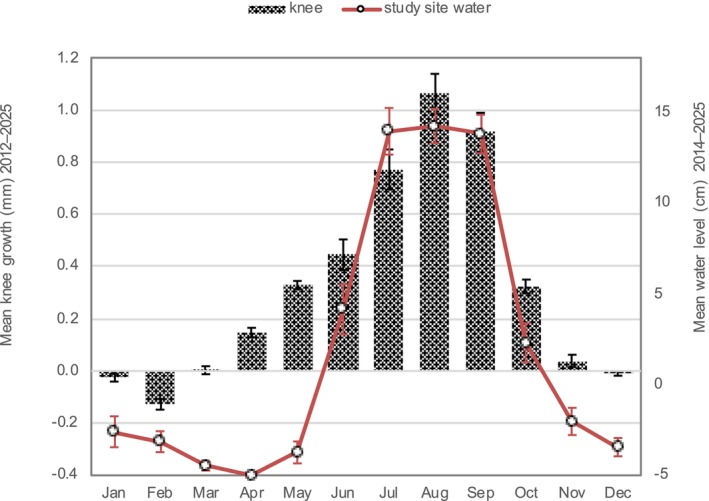
Mean knee growth and mean water level for each calendar month over 14 growing seasons. The mean knee growth (mm) is the mean of 13 or 14 monthly means (2012–2025) for 24–30 knees for each calendar month. The mean water level (cm) is the mean of 11 or 12 water level measurements (June 2014–October 2025) at the study site for each calendar month. Error bars indicate standard error.

**FIGURE 6:**
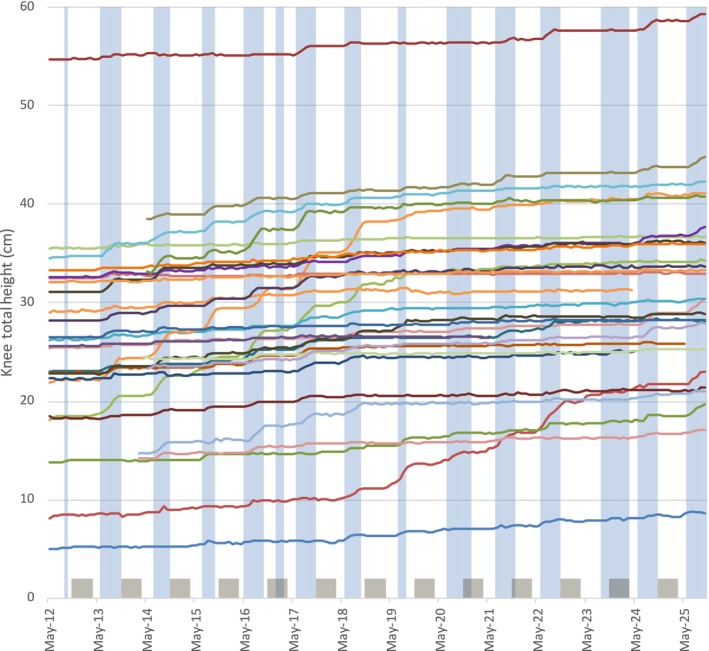
Total knee height (cm) for each knee May 2012 through October 2025. Blue vertical bars represent the presence of surface water at the study site. Knees were located at various elevations, thus the knee height does not directly indicate the knee tip's height above mean high water. Water data for May 2012–April 2014 is from the CSS database, and water data from May 2014–October 2025 is from the study site.

**FIGURE 7:**
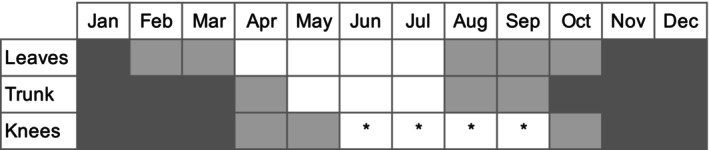
Growing seasons for cypress tree components near Corkscrew Swamp Sanctuary. Cell shading: Dark gray is dormant, light gray is transitional, and white is growing season. At this site, the potential for knee growth during April and May was undetermined since surface water did not occur during those months. Trunk data is from Duever et al. (1976). *Growth dependent upon above‐ground water levels.

Over 14 growing seasons, knee growth was found to be strictly seasonal, with very little knee tip growth from November through the end of March (Figure 4). The no‐growth hiatus for cypress knees each winter was distinct, with the monthly mean populational knee growth being close to zero during this knee dormant period (Figure 5). The growing and dormant periods are evident in line charts of individual knees, appearing as stairstep‐like height increases (Figure 6). The timing of the dormant season of study site cypress knees was compared with the dormant seasons of cypress trunks (Duever et al. 1976) and the deciduous canopy (field observations and Duever et al. 1984) in the local area (Figure 7). Although cypress trunks and leaves are waning in activity in August and September, these months were found to potentially be very active growth months for cypress knees.

Cypress knee dormancy had predictable timing, starting each November. In contrast, the knee growing season was variable each year—for the start date, the intensity, and the duration. The first month of substantial seasonal knee growth was closely correlated with the first month that southern Florida's monsoon season resulted in flooded conditions at the study site, and the month that occurred was variable each year: June (2013, 2016, 2017, 2018), July (2014, 2021, 2022, 2024, 2025), or August (2015, 2019, 2020) (Figure 4). If water levels didn't rise until late September (2012, 2023), knees did not grow that summer.

By overlapping data for knee growing seasons and data for water levels over a span of 14 years (Figures 4, 6), it is apparent that there are two requirements for seasonal cypress knee growth: (a) substantial growth occurs when water is above the surface (i.e., the subterranean cypress root system is inundated); and (b) knees grow only when the *trees* are active (i.e., knee growth is constrained by a fall/winter dormant period). At the study site, extreme weather conditions have tested these requirements, as demonstrated by the following examples.

- During two years (2012, 2023), surface water at the study site did not occur until after autumn cypress tree dormancy was in its early stages, and the cypress knees essentially did not grow during those dry summers.
- During two years (2016, 2024), wet winter El Nino conditions resulted in above‐surface water levels in January/February, and cypress knees essentially did not grow during those wet winter months.

Interestingly, there were two years when trees were unseasonably leafed out with a new flush of autumn leaves that persisted into January, yet knee growth ceased by the end of October. The new flushes of leaves were in response to Hurricane Irma (Sept. 10, 2017) and Hurricane Ian (Sept. 28, 2022), both of which denuded the cypress trees. Other than hurricanes increasing the study site water levels (see Figure 4 with indicators of water levels from three hurricanes), there was no discernible effect on knee growth due to hurricanes, and only one knee tip was damaged due to falling debris during windstorms.

In analyses to investigate long‐term correlations between knee growth data and water data, knee growth was represented as the annual growth rate of the calendar year, averaged for the population of 24–30 knees. Due to the knee dormant season, the annual growth rate and the seasonal growth rate for knees are the same. Water data, however, was restricted to the period of April 1 through October 31, when it could potentially influence knee growth. Although April through October may be the open window for cypress knee growth, in southern Florida, due to the annual dry‐down in spring, the effective knee growing season appears to be June through October.

Analyses between knee growth and water levels included not only study site water data, but also data from the nearby Corkscrew Swamp Sanctuary (CSS). The CSS water database had the advantage of daily measurements and encompassed all years of this knee study. Comparisons between the two water datasets show generally similar water level fluctuations during above‐ground water periods. During the early rainy seasons, when Corkscrew Swamp was filling, there was sometimes a lag in rising water levels at CSS. The most likely explanation for this lag is that smaller wetland water levels, such as the study site depressional swamp, are flashier and can rise and fall faster than larger wetlands such as the miles‐long cypress strand of Corkscrew Swamp. Another factor that could account for these time differences is that the core cypress strand at Corkscrew Swamp is currently having severe dry season drainage problems from downstream canals that result in delayed recovery of wet season water levels (Clem and Duever 2019).

**FIGURE 8:**
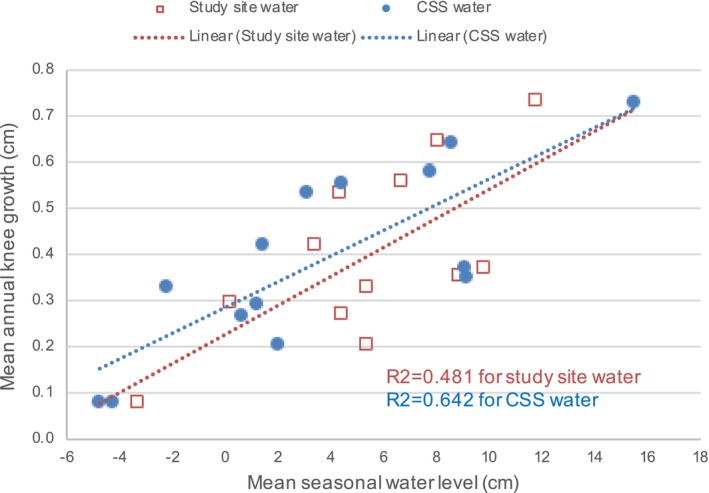
The mean annual growth of 24–30 knees from 2012 through 2025 was plotted with the mean seasonal water level from two data sources. Water levels were calibrated such that zero is the minimum level for surface water to occur at the lowest elevation knee. Seasonal water data (April 1–October 31) are means of monthly measurements for the study site, and daily measurements at Corkscrew Swamp Sanctuary (CSS) using 80.3 cm as the conversion factor. Both water datasets had a lower limit of −5 cm.

Mean annual knee growth for each of the study years was compared with mean seasonal (April 1–Oct 31) water levels (Figure 8), which produced a linear regression line that infers a positive correlation of more knee growth during wetter seasons with a fairly good fit considering the high variability of the knees themselves (*R* ^2^ = 0.481, *n* = 12, for study site water data; *R* ^2^ = 0.642, *n* = 14 for CSS water data). Mixed‐effects modeling, which accounts for repeated measurements of the same knees, implies that cypress knee growth was strongly associated with seasonal water levels (*β* = 0.032 cm knee growth per cm water, SE = 0.006, *p* < 0.001 for study site water data, and *β* = 0.028 cm knee growth per cm water, SE = 0.004, *p* < −0001 for CSS water data; Table 2). The β estimates indicate that knees grew an additional ~0.03 cm for each additional cm of mean seasonal water level, which agrees with the linear regression presented in Figure 8. Utilizing linear regressions of summary data versus mixed‐effects models for this study is debatable (see Methods). For this knee population, many individual knees were not consistent performers (i.e., fast‐growing knees were not reliably fast‐growers; Figures 3, 6). The intra‐class correlation coefficient for the mixed‐effects models, which provides information about whether or not observations from the same knee are usually more similar to each other than observations from different knees was 0.3 (on a scale of 0.0 to 1.0), indicating there is a low level of reliability of a knee's growth performance.

**TABLE 2:** Beta estimates from mixed‐effects models for knee growth.

Linear Mixed‐effect model	Fixed effect	Beta estimate	SE	*t*‐value	*p*
Annual growth model 1					
	(Intercept)	−0.099	0.086	−1.152	
	Hydroperiod (days)	0.004	0.001	7.695	< 0.001
	Number of observations: 398.				
	Correlation of fixed effects: −0.737				
	Random effects Intra‐class correlation: 0.32				
Annual growth model 2					
	(Intercept)	0.282	0.060	4.695	
	Seasonal water level CSS (cm)	0.028	0.004	7.020	< 0.001
	Number of observations: 398.				
	Correlation of fixed effects: −0.251				
	Random effects Intra‐class correlation: 0.31				
Annual growth model 3					
	(Intercept)	0.225	0.069	3.269	
	Seasonal water level Site (cm)	0.032	0.006	5.243	< 0.001
	Number of observations: 350.				
	Correlation of fixed effects: −0.477				
	Random effects Intra‐class correlation: 0.31				

Generalized linear mixed‐effect model	Fixed effect	Beta estimate	SE	*z*‐value	*p*
Growth occurrence model					
	(Intercept)	−2.1754	0.1661	−13.096	< 0.001
	Surface water yes	1.7603	0.1068	16.487	< 0.001
	Dormant season yes	−1.9535	0.1981	−9.863	< 0.001
	Surface water × dormant season interaction	−0.8129	0.3385	−2.428	0.0152
	Number of observations: 4598				

*Note:* For each model, knee identity was a random effect within the model. Annual growth models provide estimates of how much knee growth is correlated with each hydrologic condition parameter. The growth occurrence model, which relies on binomial Yes/No data, provides the probability of growth occurring under various conditions.

**FIGURE 9:**
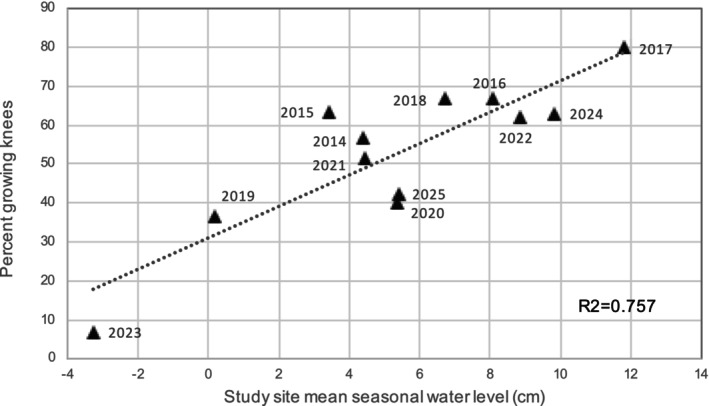
Percentage of knees that were growing, compared with mean seasonal water levels for 2014–2025. “Growing knees” is defined as knees with an annual growth rate of 0.3 cm/year or more. Seasonal water level at the study site is the mean for April 1 through October 31. Water levels were calibrated such that zero is the minimum level for surface water to occur at the lowest elevation knee. Seasonal water data (April 1–October 31) are means of monthly measurements for the study site, and daily measurements at Corkscrew Swamp Sanctuary (CSS) using 80.3 cm as the conversion factor. Both water datasets had a lower limit of −5 cm. Linear regression analysis produced an R^2^ of 0.7525 (*n* = 12) for study site water data, and (not shown) *R* ^2^ = 0.732 (*n* = 14) when plotted with CSS water data.

Years with greater amounts of knee growth were the result of a handful of knees surging with very high growth rates, plus a higher percentage of knees actively growing (Figure 3). The surging knee growth rates skew the mean for annual knee growth. An alternative comparison was done using the *percentage* of knees that grew at or above 0.3 cm/year, which raised the confidence of the positive correlation between greater knee growth and higher seasonal water levels (Figure 9; *R* ^2^ = 0.753, *n* = 12, for study site water data; *R* ^2^ = 0.731, *n* = 14, for CSS water data).

**FIGURE 10:**
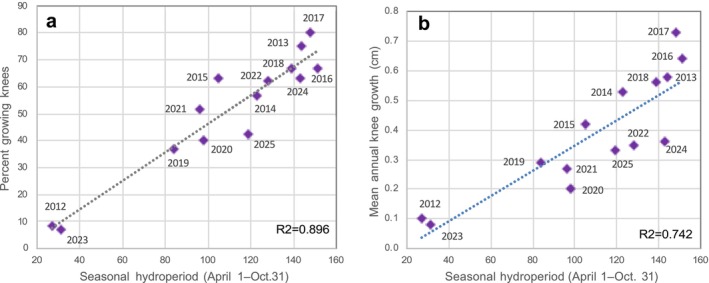
(a, b) Knee growth parameters of 24–30 knees from 2012 through 2025 plotted with the seasonal hydroperiod for the corresponding year. (a) Annual percent of growing knees (growth at ≥ 0.3 cm/year) versus hydroperiod, and (b) mean annual knee growth versus hydroperiod. The seasonal hydroperiod is the number of days between April 1 and October 31 during which field notes indicated there was surface water at the surveyed knees, or the CSS B Gauge had a value of 72 cm or higher.

From the perspective of a cypress knee, a higher seasonal water level has two components: water height and hydroperiod (number of days with water above the ground surface). Higher water results in more knee sidewall bark being inundated, and potentially some knees being overtopped, while longer hydroperiod results in more cumulative days that the subterranean root system is inundated. In a depressional swamp or strand, water height cannot effectively be segregated from hydroperiod since higher water levels contribute to longer hydroperiods. Nevertheless, knee growth characteristics were compared with seasonal hydroperiod length between April 1 and Oct. 31. The hydroperiod for each year at the study site was estimated primarily based on field notes indicating the date of seasonal onset of surface water at the knees; then data gaps were filled using CSS water data employing the early‐season conversion factor (see Methods). Plotting the percent of growing knees with the seasonal hydroperiod of the corresponding year produced a linear regression line with *R* ^2^ = 0.896 (*n* = 14) (Figure 10a), indicating a very strong correlation between longer hydroperiods and more growing knees. Similarly, comparing mean annual knee growth with seasonal hydroperiods generated a regression line of *R* ^2^ = 0.742 (*n* = 14) (Figure 10b). Mixed‐effects modeling also supports a positive correlation between cypress knee annual growth and hydroperiod (*β* = 0.004 cm growth per day, SE = 0.001, *p* < 0.001; Table 2). This relationship corresponds to approximately 0.04 cm knee growth for each additional 10 days of hydroperiod. Although hydroperiod appears to explain much of the trend of greater knee growth during wetter years, water height certainly influences knee growth. For example, looking at 2016 and 2017, both had a similar hydroperiod (Figure 10), but 2017 had a much higher water level than 2016 (Figure 9), and much greater knee growth both in terms of cm/yr. growth and the percentage of knees that were growing. An additional consideration in interpreting the effects of hydrology is that the relationship may be nonlinear or involve threshold responses.

**FIGURE 11:**
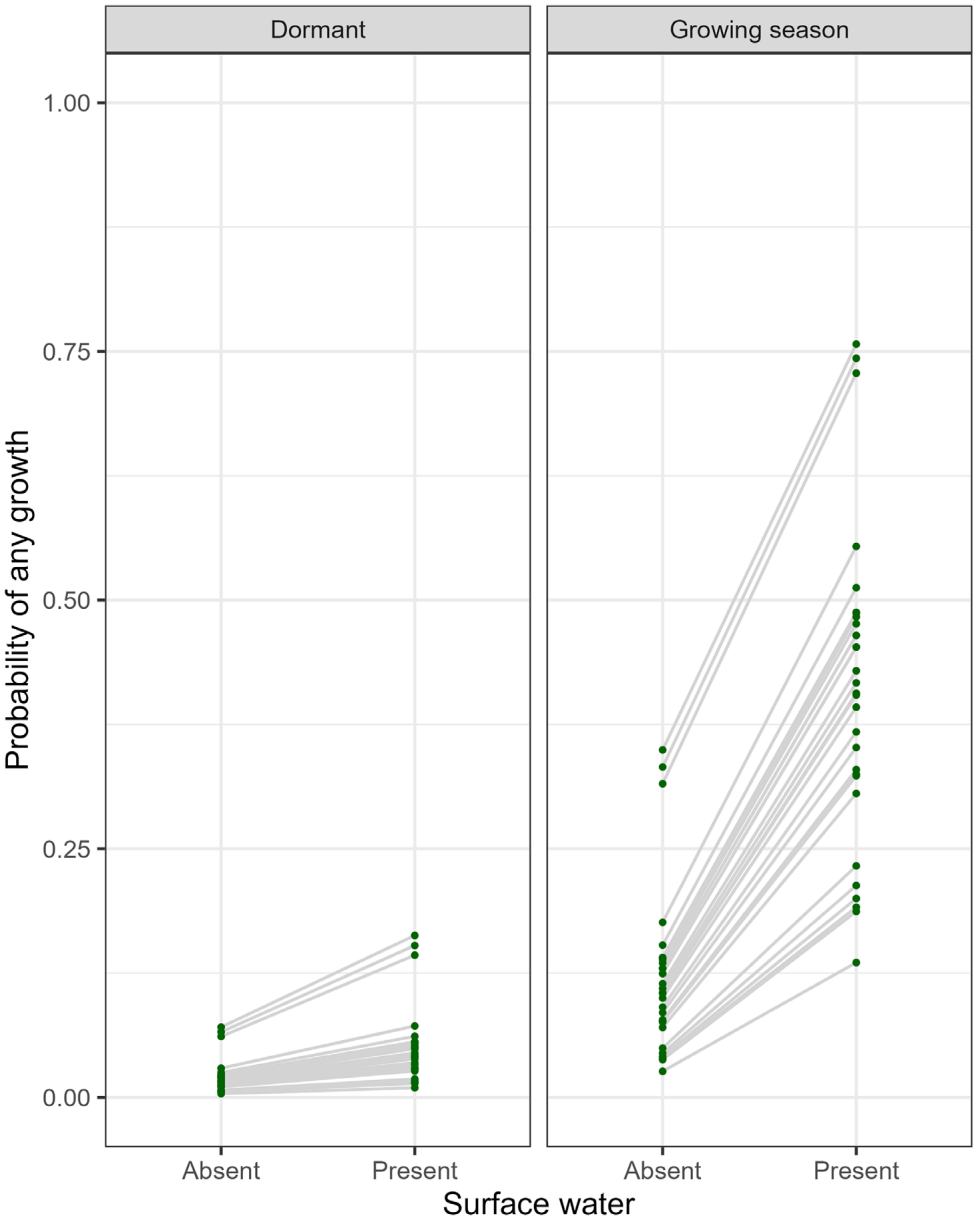
Probability of knee growth in the absence/presence of surface water during growing season (April–October) and dormant season (November–March). Probabilities were determined via a generalized linear mixed‐effects model with a binomial error distribution, which was calculated based on each of the 30 knees' monthly growth data, considering the four conditions, over the duration of the 14‐year survey. Surface water presence/absence was from Corkscrew Swamp Sanctuary data from June 2012 through March 2014, and study site water data from April 2014 through October 2025.

Mixed‐effects modeling of monthly knee growth data provides statistical support for the presence of surface water strongly increasing the probability of measurable knee growth (*β* = 1.76, SE = 0.107, *p* < 0.001; Table 2). The probability of knee growth was very low during the dormant season, November–March (*β* = −1.956, SE = 0.198, *p* < 0.001). The significant interaction term (*β* = −0.8129, SE = 0.339, *p* = 0.015; Table 2) suggests that during the uncommon condition of having dormant season months with surface water, the stimulatory effect of surface water on knee growth was much weaker than during the growing season, but still present. The interaction model had 89% of the weight and an AAIC of 4.21 over the model without an interaction. Figure 11 provides a graphic display of the mixed‐effect model probability predictions for each of these conditions for each knee.

For this population of mature knees, there was no correlation (*R* ^2^ = 0.006) between the 14‐year mean growth rate for each knee and its height above or below mean high water. Three knees (10%) were inundated during high water at the start of this study, and one of those knees (#302) grew above mean high water with a surge of growth during the latter part of the study period. For this population, there was no correlation (*R* ^2^ = 0.080) between individual knee growth rates and the knee's elevation (i.e., depth below mean high water). Of various knee architectures (cone‐shaped, or merged with other knees, or root‐loop knees), none were correlated with greater growth rates.

## 4 Discussion

Monthly monitoring of 30 cypress knees throughout 14 years has revealed two patterns: (1) knee growth occurs seasonally with a dormant period from November through March, and (2) the magnitude of seasonal knee tip growth increases with longer hydroperiods and higher water levels. From the data generated by this study, it is apparent that these conclusions would not have been feasible if monitoring a small number of knees, nor a short time span. Following these 30 knees over the years revealed how individual knee growth can be quiescent, or intermittent, or include surge phases (Figures 3, 6). “Growth”, as measured by knee tip extension, is oversimplified and fails to capture knee volume expansion in girth or at bulges. New field technologies such as LiDAR applications (e.g., Martinez et al. 2024; Tajudeen et al. 2025) may assist in improving knee size measurements in future studies. It is difficult to overemphasize the variability of cypress knees. Beyond their obvious variability in size, shape, and density per hectare, each cypress forest location has different hydrological and ecological conditions that may result in knee assemblages that do not conform to patterns observed elsewhere.

This study in a depressional cypress dome generated a mean knee growth rate of 0.4 cm/year. That growth rate was twice the growth rate of old‐growth cypress strand knees nearby (Bonness et al. 2013). At both study sites, 90% of knees are above mean high water, with all knees being relatively mature (none had recently emerged from the ground). Older cypress tree trunks grow slower than younger tree trunks (Duever et al. 1976), and a declining growth rate as trees age could also apply to roots. Delving further into the data that generated the old‐growth knee rate of 0.2 cm/year (Bonness et al. 2013), the data can be subdivided by location and age‐class: 0.16 cm/year knee growth for cypress strand interior plots (200–400 year‐old trees; *n* = 43 knees), and 0.27 cm/year knee growth for strand outer‐edge plots (100–200 year‐old trees; *n* = 27 knees). Although the cypress dome trees have not been aged, the trees have a considerably smaller diameter than those of the old‐growth cypress plots and may be closer to a median age of 100 years (Michael Duever, personal observation). These data imply a trend of younger trees having faster‐growing knees.

At the study site during the dry months of April and May, some knees had small amounts of growth with a hint of bark expansion, as if prepped for a growth event. This raises questions about knee growth patterns in other parts of the cypress range that have springtime flood events. Likewise, it raises questions about knee growth patterns along rivers, where water levels can fluctuate intensely, and it may be an annual occurrence for most knees of a population to be briefly submerged.

Results from this study imply that it is advantageous for cypress trees to have more expanding knee tips during wetter conditions. Considering that 90% of surveyed knee tips were above high water at the start of the study, it raises the question: Is there a purpose for tip growth other than simply getting higher above water? Indeed, most knees within natural areas are mature knees (decades or centuries old) with their tips well above high water, and yet many continue to put on fresh tip growth. Anatomically, new tip growth has little or no outer bark, plus minor air spaces (Rogers 2021; Yamamoto 1992), thereby reducing the diffusional resistance of gases to the inner bark and the vascular system. *Taxodium* roots, unlike many species that are adapted to growing with inundated roots, do not have aerenchyma tissue (Penfound 1934) with a network of intercellular air spaces that allows open‐vent passage of gases between organs. Despite the lack of aerenchyma tissue, methane appears to move from roots to knees to the atmosphere at flux rates sometimes greater than soil‐to‐atmosphere (Martinez et al. 2024), thereby implying that knees can serve as conduits for gas exchange. If cypress knees are acting as pneumatophores (facilitating oxygenation of the subterranean root system), oxygen could diffuse through the porosity of the outer layers and subsequently be transported through the vasculature as “oxygenated sap” (Rogers 2021; Shaler 1887). Although older knee bark likely allows oxygen to reach the inner bark, new tip growth could temporarily augment oxygenation of the inner bark (one less layer to permeate) while also expanding the above‐water knee surface area. In other words, cypress knees breathe, and they may breathe more easily with newly expanding tips. To the extent that inhalations of knees go beyond the knees themselves, into the root network below, remains to be demonstrated in a living tree.

Knees are not islands. Although knees were measured as “individual knees”, they are connected to an unseen, unmonitored subterranean root network. The physiological condition of the inundated root *system*, as water levels and resources fluctuate, influences hormone distribution and hormone receptor activation, which subsequently affects which knees grow and how much. A notably slow growth rate of ≤ 0.1 cm/year was recorded for five knees (17%) over the 14‐year study. Very slow growth was also recorded for five knees (only two of which were also 14‐year slow‐growers) during the extremely wet year of 2017 (Figure 3). Although the hydrologic environment appears to be a significant driver, it does not entirely account for knee growth rates. An interesting example is seen with knees #301 and #302, which are inches apart and protrude from the same lateral root (Appendix S1). Despite sharing the same vascular system and having a similar environment, knee #302 surged (1.06 cm/year rate), while knee #301 had lackluster growth (0.26 cm/year rate) and is overtopped by water during most wet seasons. Perhaps knee #302's height and fresh tip growth were sufficient for the physiological requisites of the nearby root system. A better understanding of why some knees grow, while others do not grow, would likely involve a more expansive understanding of physiological conditions (including carbohydrate metrics) within the root system and rhizosphere below.

Not all knees in a population grow when the forest floor is flooded, and not all growing knees have a flooded base. It is not unusual to find growing knees within a higher substrate that occurs adjacent to a cypress tree's inundated root system. This is particularly common in southwestern Florida along former logging railroad beds (“trams”) from the 1950's cypress logging operations. In shallow water conditions, cypress trees have a lower “cost” of growing a knee with a tip above water. On the other hand, the greatest benefits of having knee tips above water may be realized in deeper water with longer anoxic conditions. However, it can take years or decades for a new knee to grow to a suitable height to generate benefits during the wet season. At some point, the cost/benefit ratio skews the odds against growing a knee in very deep water. This cost/benefit conundrum for knees may help to explain Yamamoto's (1992) seemingly contradictory finding of higher knee density in shallower water. At this study site, the cypress dome, knee density is higher in the shallower outskirts, although the outskirt knees are generally smaller.

Woody plant parts are typically multi‐functional, and this is likely also true for cypress knees. Whatever the knee functions are, there is little doubt they are associated with the knee tip being exposed to air during the wet season. The only undisputed role of knees is starch storage (Brown and Montz 1986). Starch storage is a major function of roots in general, but why cypress trees have a root organ packed with carbohydrate reserves that is located above‐ground and partially above high water raises questions. If knees have a dual purpose of carbohydrate storage and oxygen intake, then energy provided by aerobic respiration within the knee would be readily available for starch resource management (assimilation, translocation, utilization), while also being co‐located with a putative pump station for oxygenated sap—a metabolic activity center with food, air, and transport combined (personal communication G.K. Rogers). The correlations between hydrology and knee tip growth identified by this study do not directly establish physiological function. Functional interpretations herein remain inferential in the absence of direct physiological measurements and do not exclude alternative explanations.

The mechanical support function attributed to knees may be more appropriately applied to a cluster of anchoring roots typically found below knees that sprawl or dive vertically below the knee (Figure 1f). The anchoring roots, which are readily visible if substrate is eroded, create a spot‐weld to assist in mechanical support for a tree that dominates soggy soils in forests prone to hurricanes. Cypress trees are more likely to have upper tree parts snap off than to fatally tip over during a hurricane (Duever and McCollom 1993). At the study site, this has been true during two major hurricanes.

## Author Contributions

**Maureen S. Bonness:** conceptualization (lead), data curation (lead), formal analysis (lead), investigation (lead), methodology (lead), resources (lead), software (lead), validation (lead), visualization (lead), writing – original draft (lead), writing – review and editing (lead).

## Conflicts of Interest

The author declares no conflicts of interest.

## Supporting information


**Appendix S1:** ece373853‐sup‐0001‐AppendixS1.pdf.

## Data Availability

Data supporting this article are available as Supporting Information.
