# Supplementary material for: Cypress (Taxodium) Knee Seasonal Growth Is Stimulated by Flood Water Levels and Constrained by the Tree Dormant Season: A 14‐Year Study
Source: Ecol Evol. 2026 Jun 15;16(6):e73853. doi: 10.1002/ece3.73853 (PMC13268810; doi:10.1002/ece3.73853)
Supplement: Supplementary file 1 — Appendix S1: ece373853‐sup‐0001‐AppendixS1.pdf. [file ECE3-16-e73853-s001.pdf]

Knees #301–321 photo comparison between 2012 and 2026

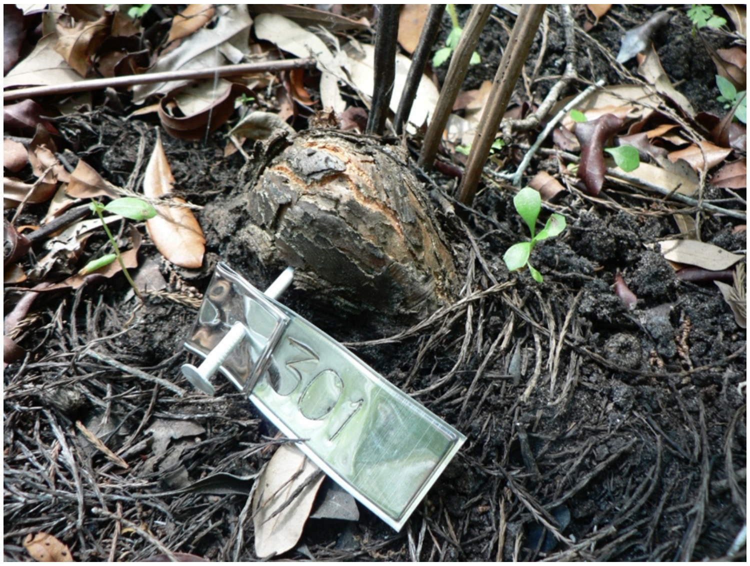

#301 in 2012 (total height 5.0 cm)

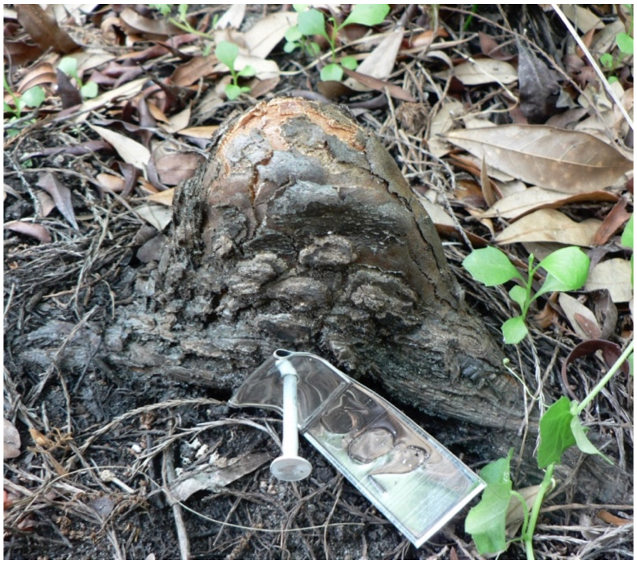

#302 in 2012 (total height 8.1 cm)

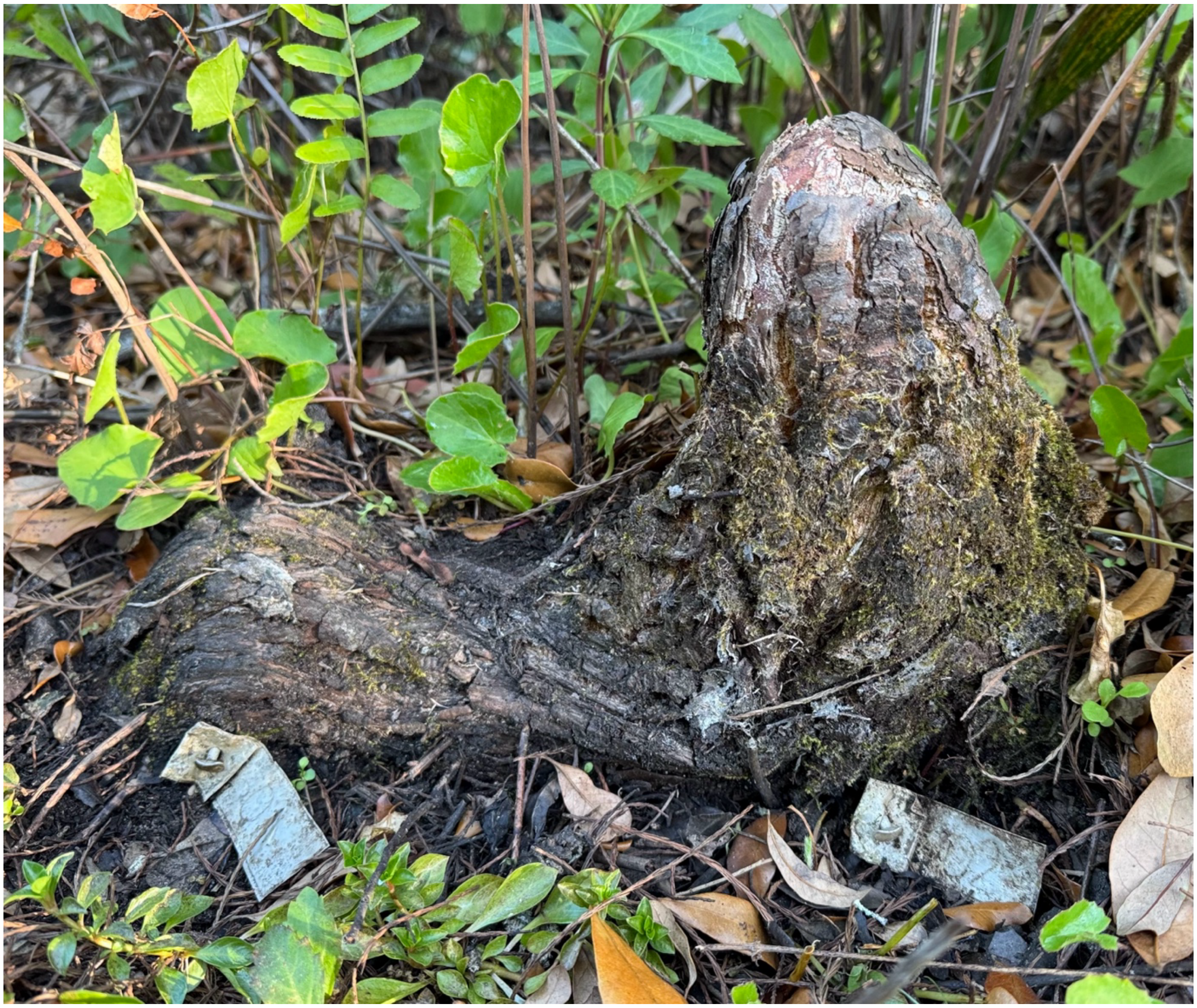

Knees #301 (left) in 2026 (3.6 cm taller) and #302 (right) in 2026 (14.9 cm taller).
Both protrude from the same lateral root.

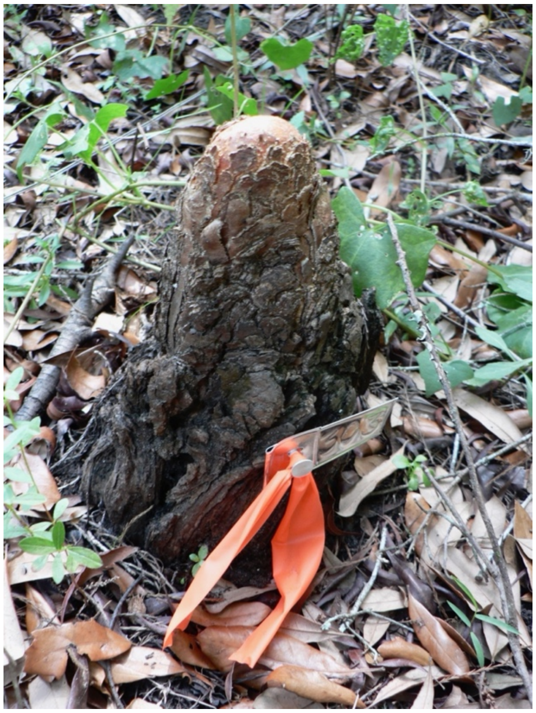

#303 in 2012 (total height 18.1 cm)

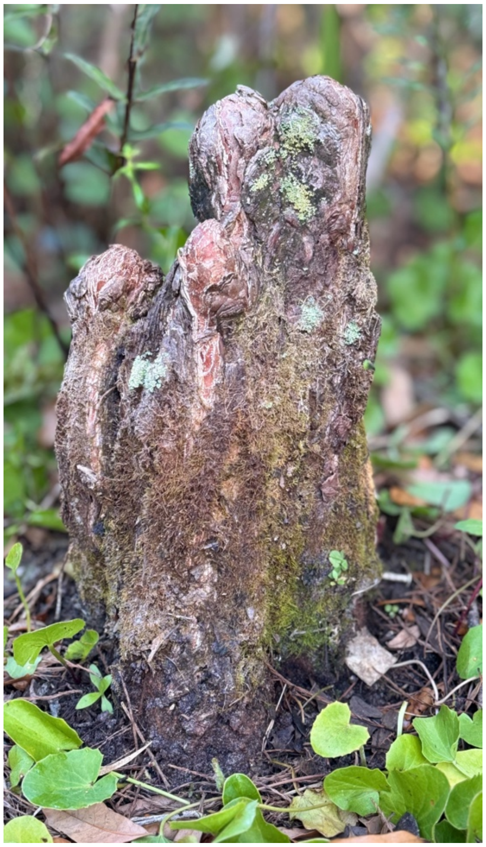

#303 in 2026 (16.2 cm taller) has 4 tips

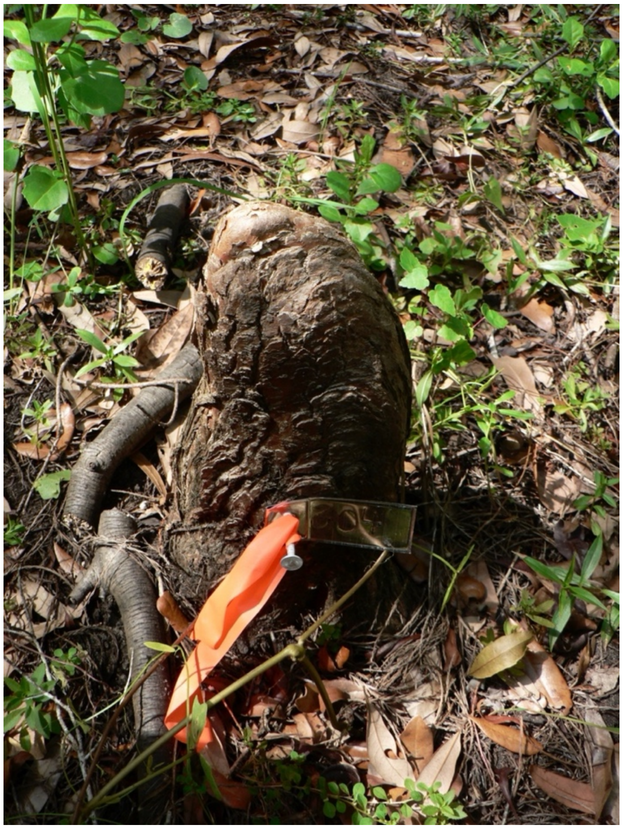

#304 in 2012 (total height 25.4 cm)

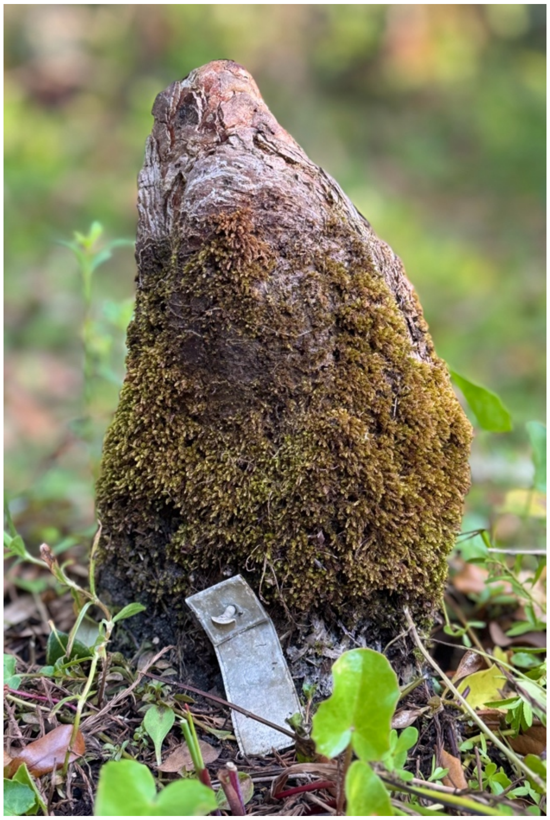

#304 in 2026 (5.0 cm taller)

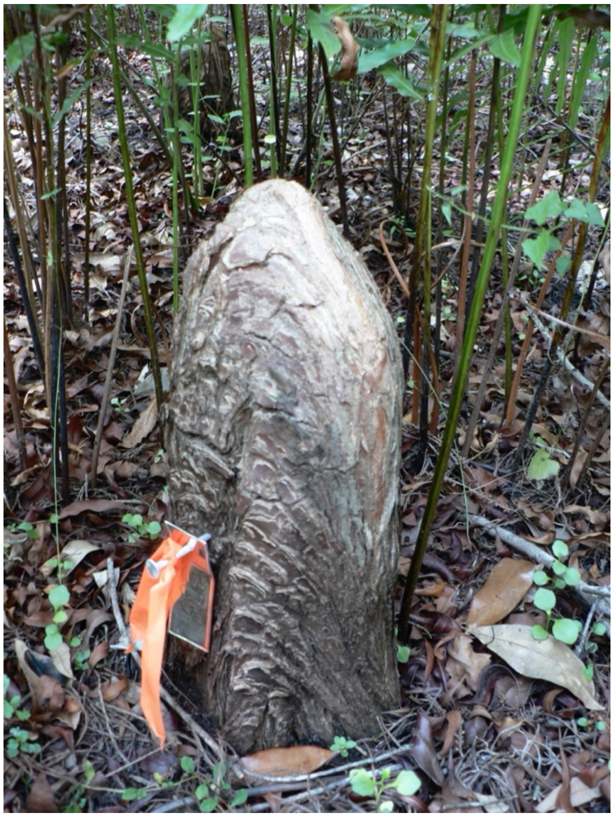

#305 in 2012 (total height 26.2 cm)

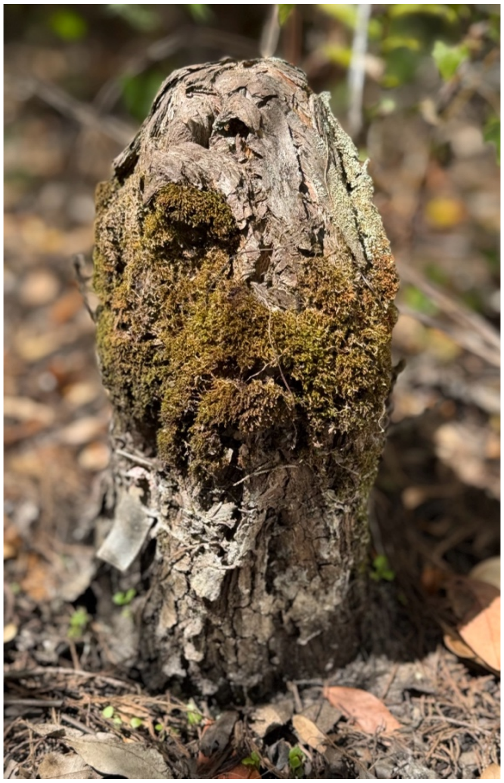

#305 in 2026 (4.2 cm taller)

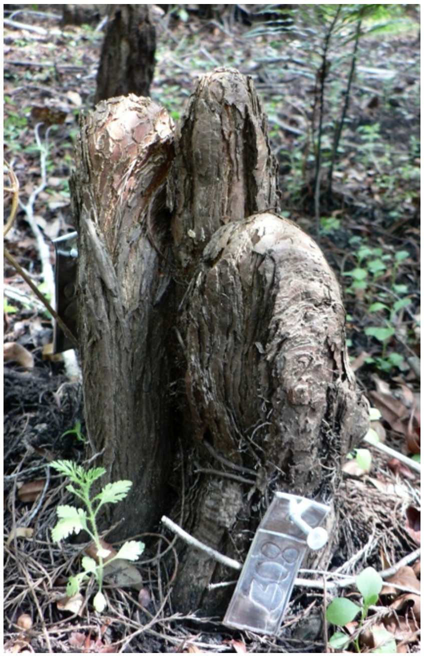

#306 (L) in 2012 (total height 21.9 cm),
#307 (mid) in 2012 (total height 22.3 cm),
#308 (R) in 2012 (total height 18.5 cm)

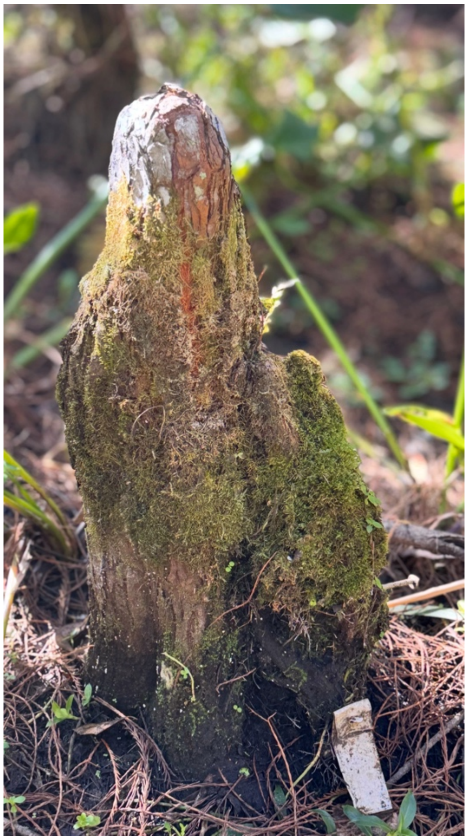

#306 (L) in 2026 (19.2 cm taller),
#307 (mid) In 2026 (2.8 cm taller),
#308 (R) in 2026 (2.9 cm taller)

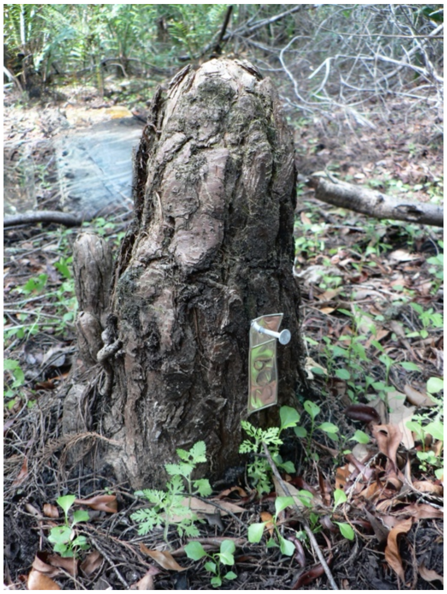

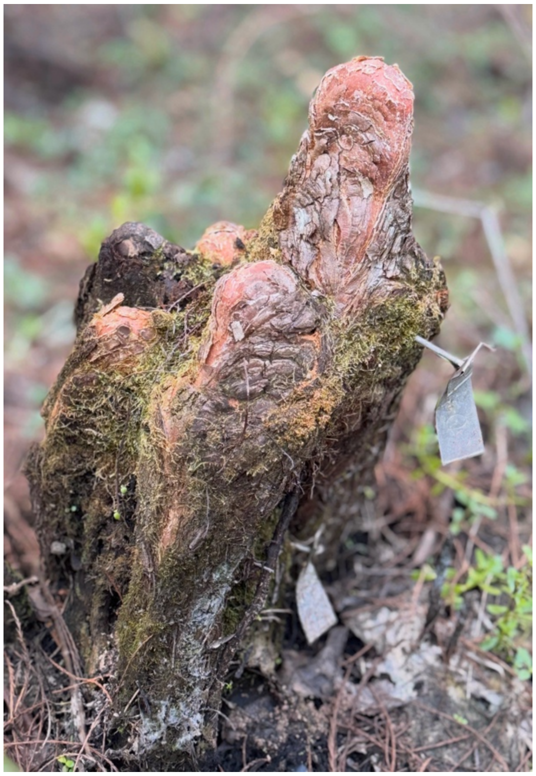

#309 in 2012 (total height 29.0 cm)

#309 in 2026 is the brown (dead) tip in upper left. Root-loop/knee #309b in 2026 has 4 growing (orange) tips

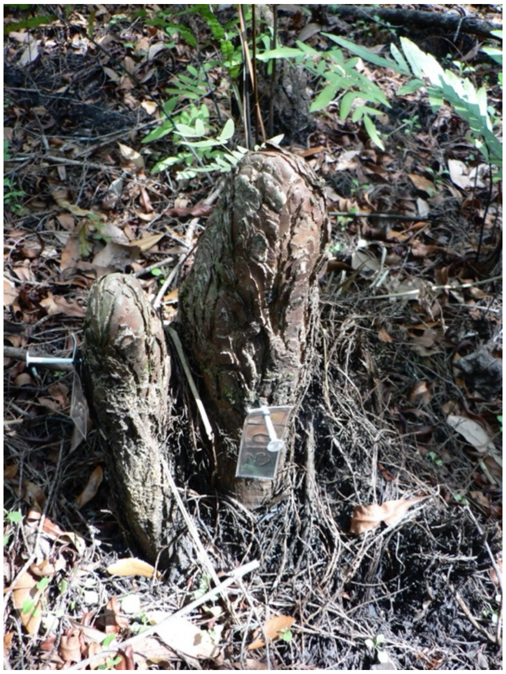

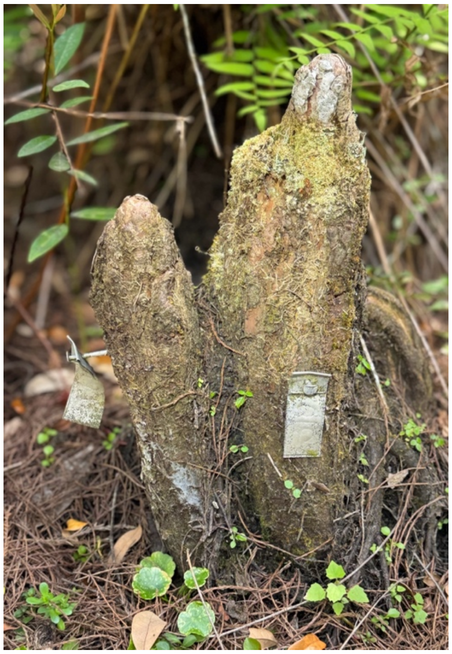

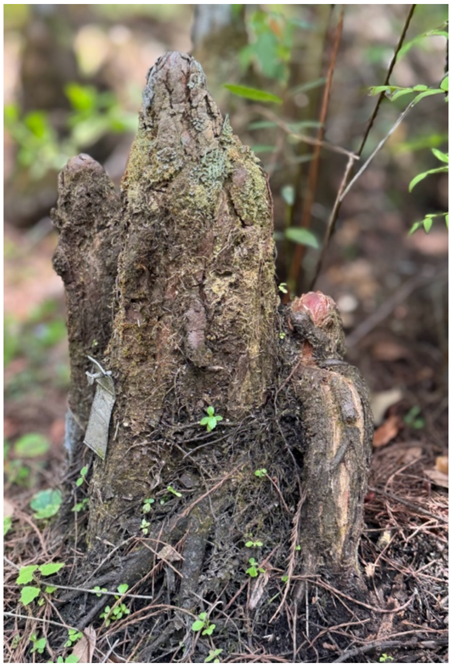

#310 (L) in 2012 (total height 28.2 cm)
#311 (R) in 2012 (total height 23.0 cm)

#310 (L) in 2026 (5.5 cm taller)
#311 in 2026 (5.3 cm taller)

In 2026 #311 has an adjacent root loop on the backside.

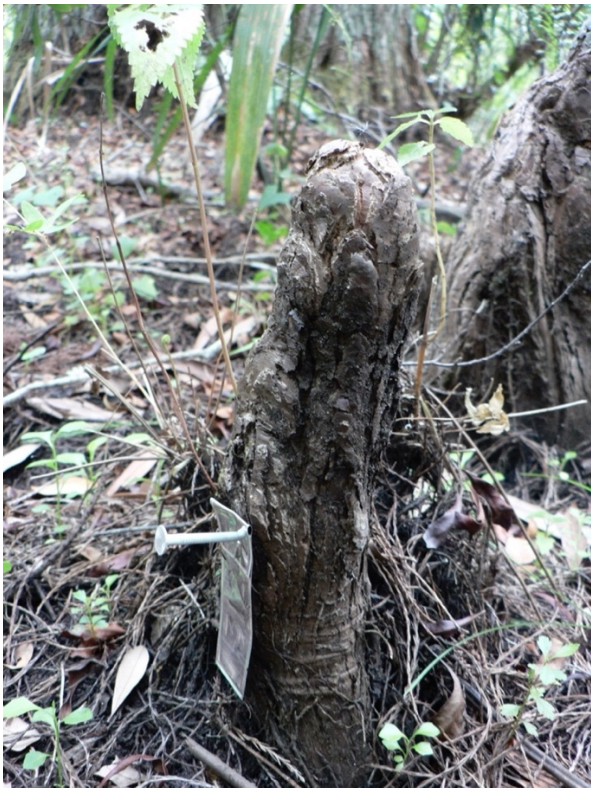

#312 in 2012 (total height 22.9 cm)

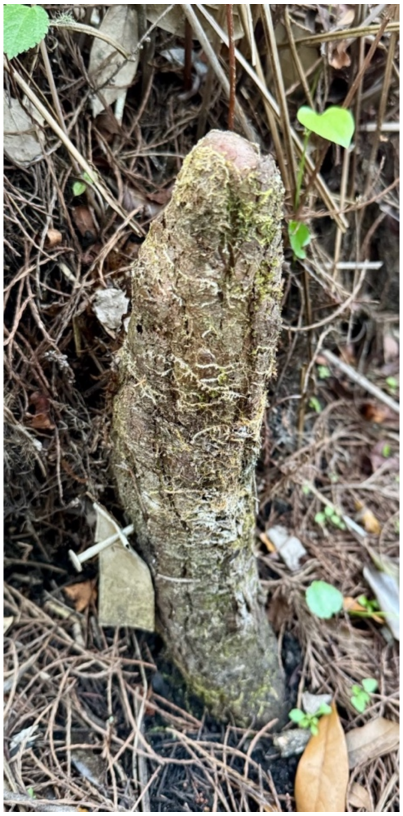

#312 in 2026 (3.0 cm taller) has bald tip

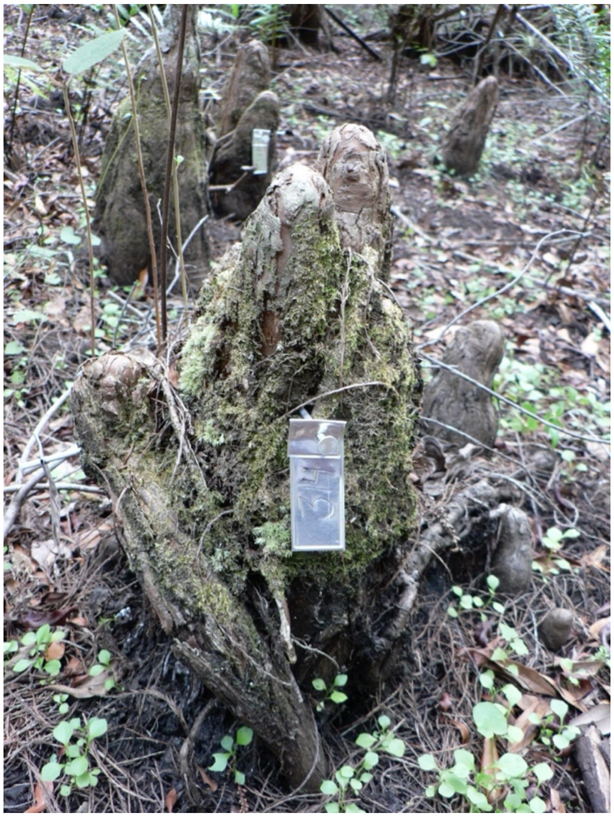

#313 (L) in 2012 (total height 22.8 cm),
#314 (middle) in 2012 (total height 32.5 cm),
#315 (R) in 2012 (total height 35.5 cm)
#313 is a root-loop/knee

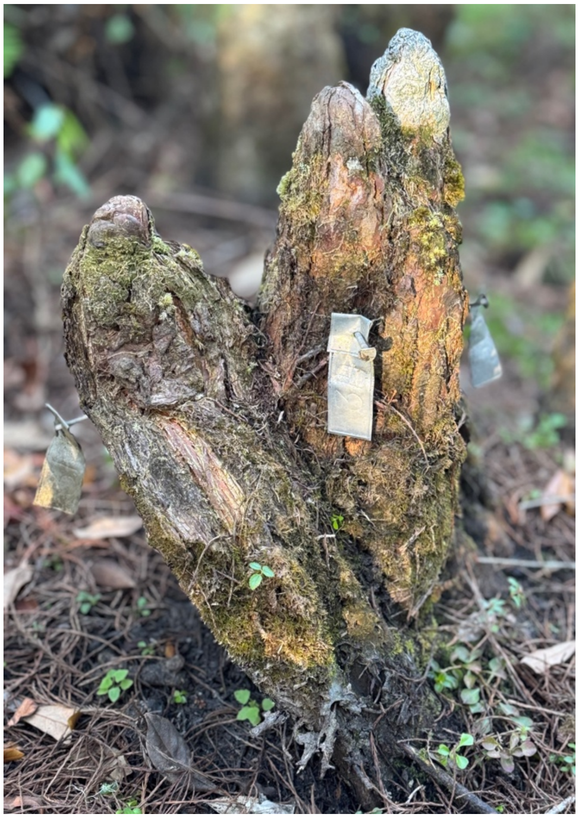

#313 (L), in 2026 (6.1 cm taller),
#314 (middle) in 2026 (0.5 cm taller),
#315 (R) in 2026 (1.2 cm taller)

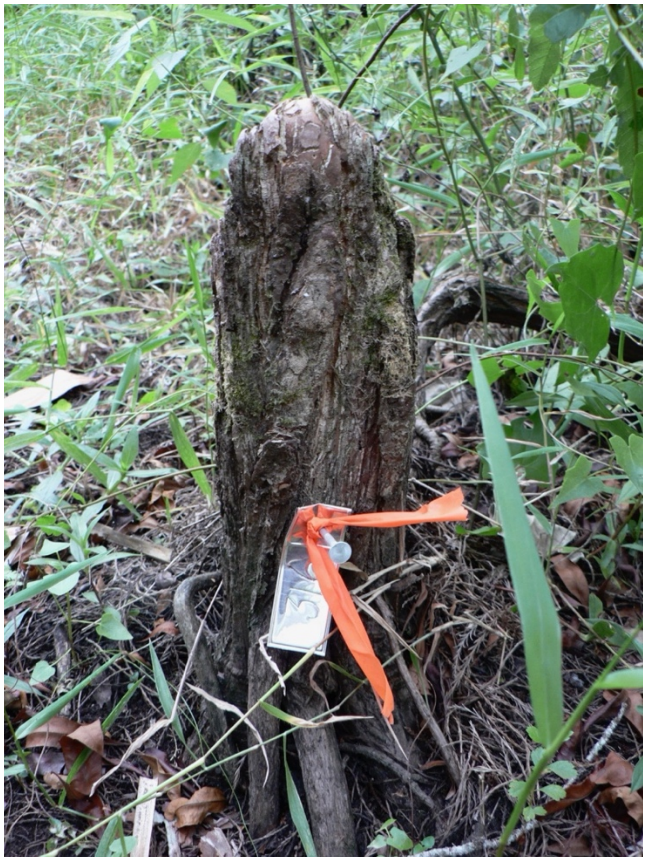

#316 in 2012 (total height 32.6 cm)

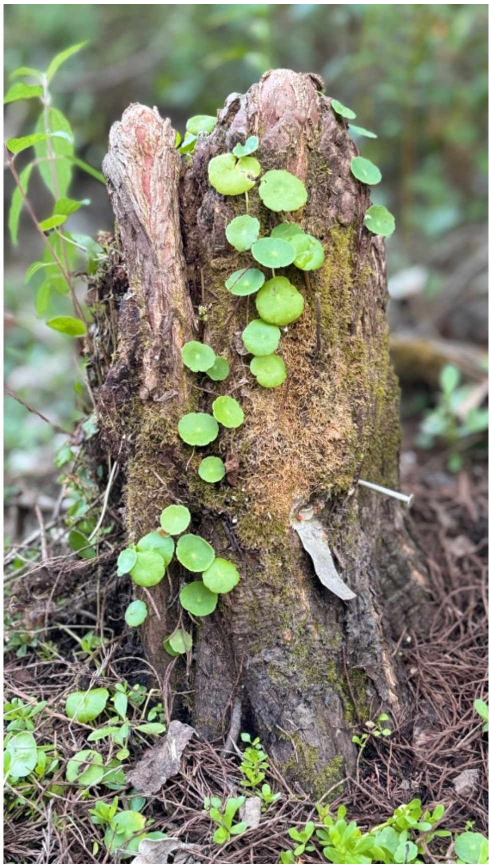

#316 in 2026 (5.1 cm taller) has a root loop
Nearly as tall as #316 tip

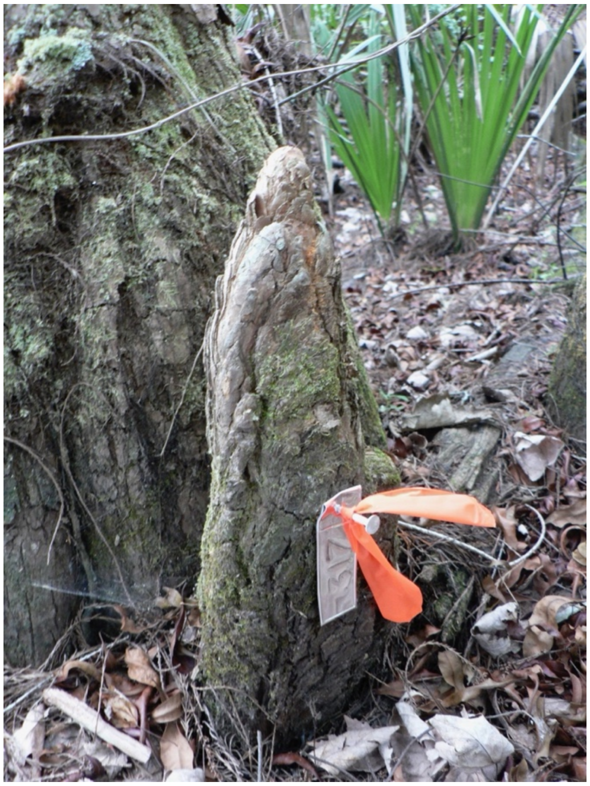

#317 in 2012 (total height 34.5 cm)

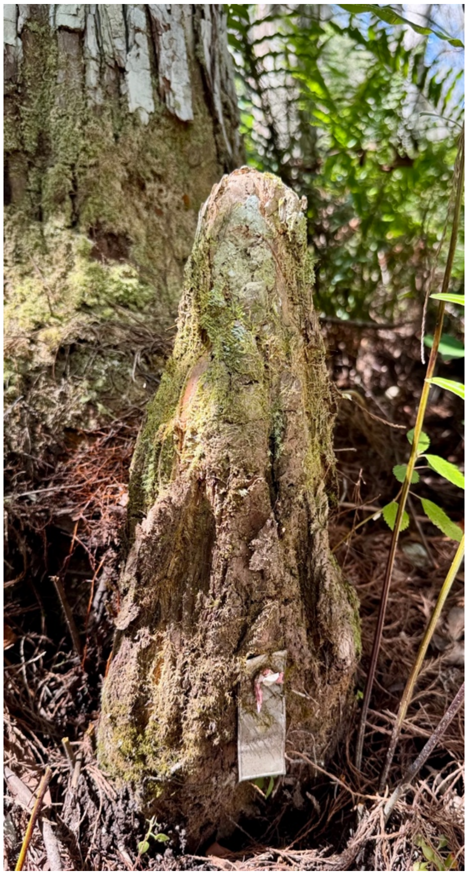

#317 in 2026 (7.8 cm taller)

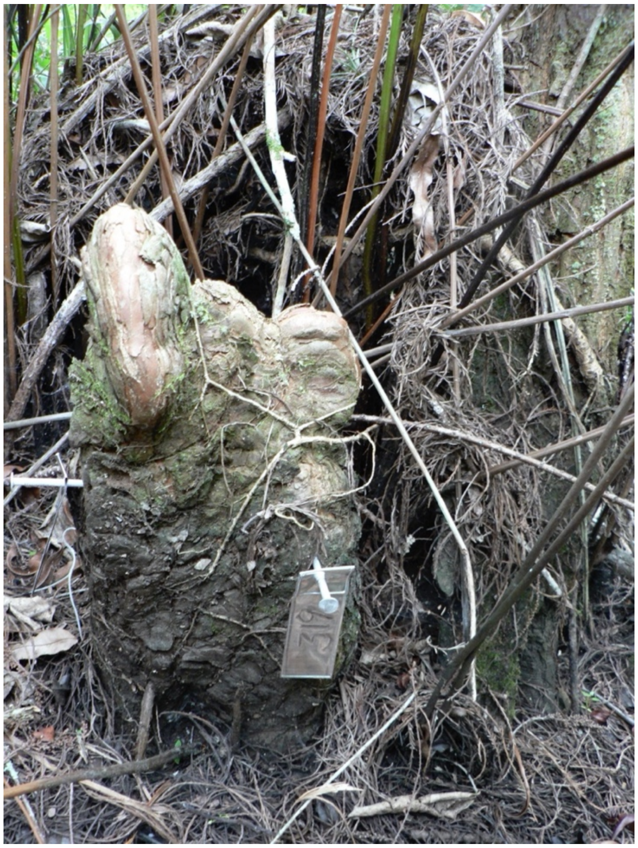

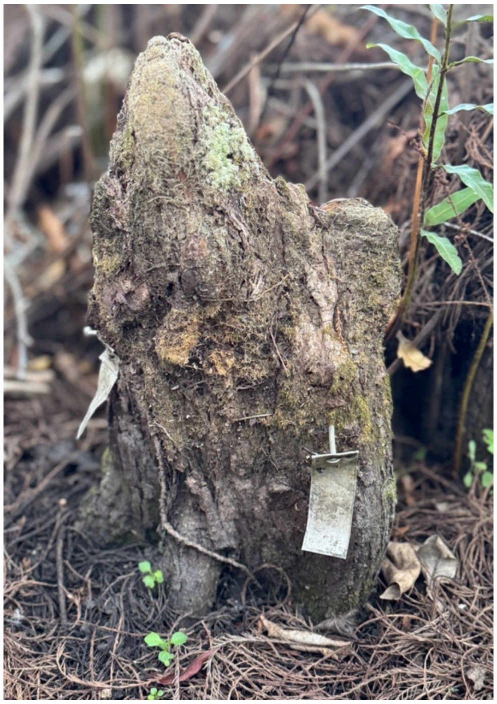

#318 (L) in 2012 (total height 31.1 cm),
#319 (R) in 2012 (total height 26.5 cm)

#318 (L) in 2025 (5.0 cm taller),
#319 (R) in 2026 (1.6 cm taller)

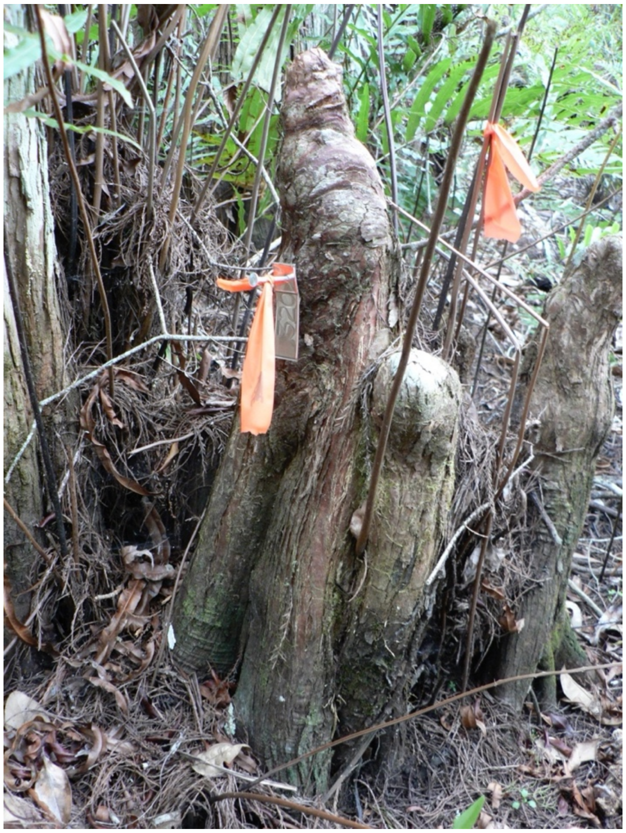

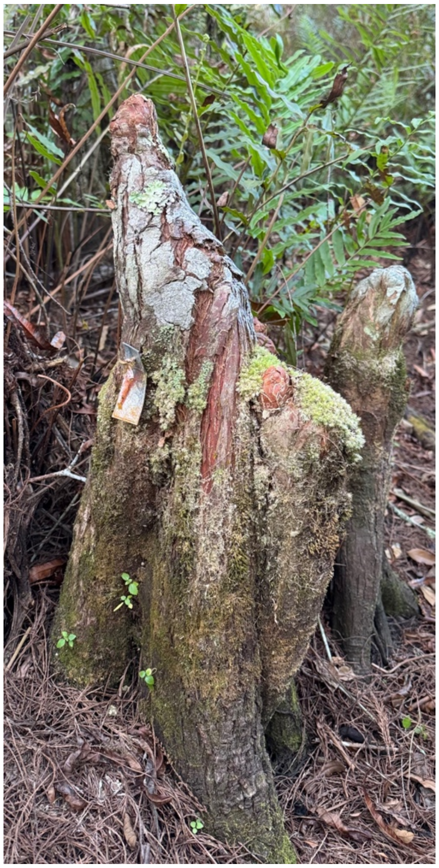

#320 in 2012 (total height 54.7 cm)

#320 in 2026 (4.6 cm taller)

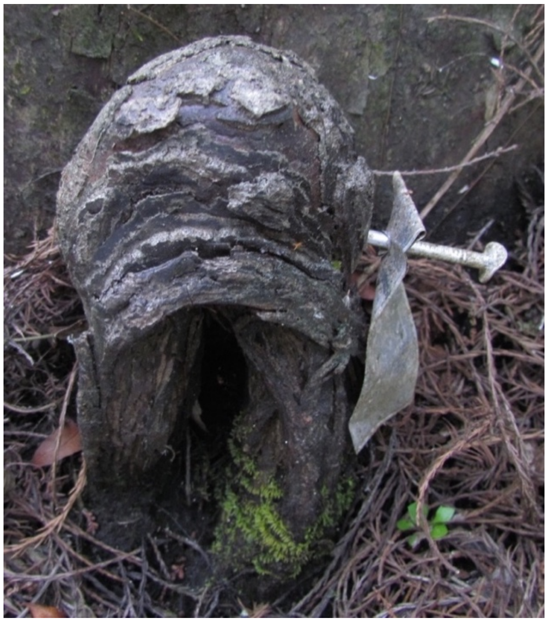

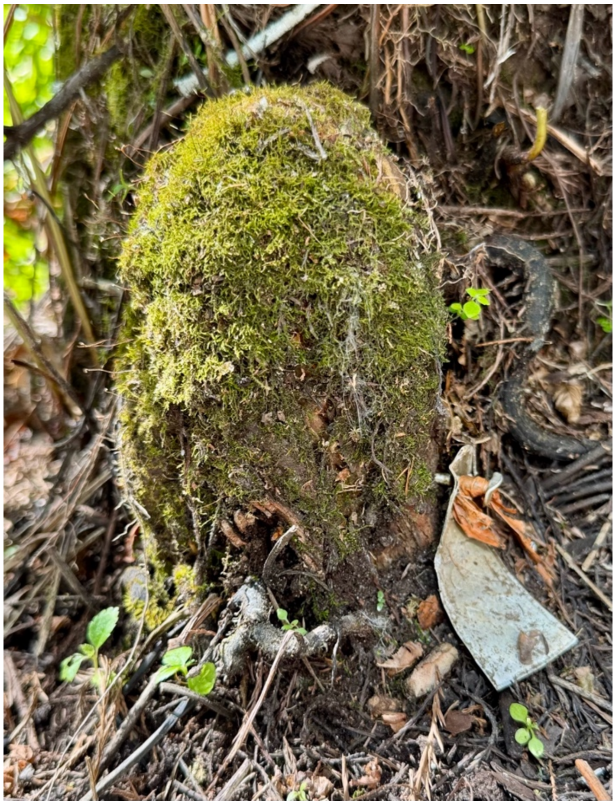

#321 in 2012 (total height 13.8 cm)
#321 is a root-loop/knee,

#321 in 2026 (5.9 cm taller)
